# Y-chromosome diversity suggests southern origin and Paleolithic backwave migration of Austro-Asiatic speakers from eastern Asia to the Indian subcontinent

**DOI:** 10.1038/srep15486

**Published:** 2015-10-20

**Authors:** Xiaoming Zhang, Shiyu Liao, Xuebin Qi, Jiewei Liu, Jatupol Kampuansai, Hui Zhang, Zhaohui Yang, Bun Serey, Tuot Sovannary, Long Bunnath, Hong Seang Aun, Ham Samnom, Daoroong Kangwanpong, Hong Shi, Bing Su

**Affiliations:** 1 State Key Laboratory of Genetic Resources and Evolution, Kunming Institute of Zoology, Chinese Academy of Sciences, Kunming 650223, China; 2 School of Life Sciences, Anhui University, Hefei 230039, China; 3 Institute of Primate Translational Medicine, Kunming University of Science and Technology, Kunming 650500, China; 4 Yunnan Key Laboratory of Primate Biomedical Research, Kunming 650500, China; 5 Department of Biology, Faculty of Science, Chiang Mai University, Chiang Mai 50200, Thailand; 6 Department of Geography and Land Management, Royal University of Phnom Penh, Phnom Penh 12000, Cambodia; 7 Capacity Development Facilitator for Handicap International Federation and Freelance Research, Battambang 02358, Cambodia; 8 Kunming College of Life Science, University of Chinese Academy of Sciences, Beijing 100101, China

## Abstract

Analyses of an Asian-specific Y-chromosome lineage (O2a1-M95)—the dominant paternal lineage in Austro-Asiatic (AA) speaking populations, who are found on both sides of the Bay of Bengal—led to two competing hypothesis of this group’s geographic origin and migratory routes. One hypothesis posits the origin of the AA speakers in India and an eastward dispersal to Southeast Asia, while the other places an origin in Southeast Asia with westward dispersal to India. Here, we collected samples of AA-speaking populations from mainland Southeast Asia (MSEA) and southern China, and genotyped 16 Y-STRs of 343 males who belong to the O2a1-M95 lineage. Combining our samples with previous data, we analyzed both the Y-chromosome and mtDNA diversities. We generated a comprehensive picture of the O2a1-M95 lineage in Asia. We demonstrated that the O2a1-M95 lineage originated in the southern East Asia among the Daic-speaking populations ~20–40 thousand years ago and then dispersed southward to Southeast Asia after the Last Glacial Maximum before moving westward to the Indian subcontinent. This migration resulted in the current distribution of this Y-chromosome lineage in the AA-speaking populations. Further analysis of mtDNA diversity showed a different pattern, supporting a previously proposed sex-biased admixture of the AA-speaking populations in India.

There is a broad consensus that modern humans originated in Africa and then migrated to Asia along a coastal route by way of the Indian subcontinent as early as 60 thousand years ago (KYA)^1^,^2^,^3^,^4^,^5^,^6^,^7^. However, the later dispersion of this ancestral population across Asia is far less clear. Linguistic analyses have grouped Asian populations across eight language families in eastern Asia and South Asia: Altaic, Sino-Tibetan (ST, split into Han and Tibeto-Burman (TB) sub-branches), Daic, Hmong-Mien (HM), Austro-Asiatic (AA), Austronesian (AU), Dravidian (DR) and Indo-European (IE). With wide distribution in mainland China and Siberia, both Altaic and ST form two northern language families, DR and IE comprise the two main language families of the Indian subcontinent, while Daic, HM, AA and AU make up the southern language families that are primarily distributed in southern China and Southeast Asia.

Trying to use linguistic families to map out the origin and migration patterns of human populations in Asia has resulted in far less consensus. For example, of the southern language families, AA has a somewhat unique geographic distribution, with a wide distribution not only in southern China and Southeast Asia, but also in India. Subsequently, AA is the eighth largest language family in the world in terms of population size (104 millions)^8^ with two major branches: Munda in eastern, northeastern and central India and Mon-Khmer, which stretches from northeastern India to the Andaman-Nicobar islands, Malay Peninsula and vast Mekong delta in MSEA. AA is the first language of many ethnic groups in Cambodia, Vietnam, Laos, Thailand, Burma and Malaysia, and serves as the main official language in Cambodia and Vietnam. Taking these realities into account, decades of research has resulted in long-standing debate about the geographic origin and prehistoric migratory route of the AA-speaking populations.

Similarly, analysis of genetic data to characterize the origin and migration history of AA-speaking populations has led to two rival hypotheses^9^,^10^,^11^,^12^,^13^,^14^,^15^. Data from the maternal lineage (mtDNA) makes a clear distinction between Munda-speakers in India and Mon-Khmer speakers in Southeast Asia, with a lack of shared mtDNA haplogroups^9^,^15^,^16^,^17^. By contrast, data from the paternal lineage (Y-chromosome) indicates a shared Asian-specific haplogroup (O2a1-M95) between the AA speakers from India (66.44% on average) and from Southeast Asia (56.55% on average)^9^,^10^,^12^,^13^,^18^. Given the relatively young age (<10 KYA) of the O2a1-M95 lineage estimated from the Y-chromosome short tandem repeats (Y-STRs) variation in India, the migratory route of the AA speakers would likely begin in Southeast Asia and then move to India^11^,^12^. However, the high mtDNA haplotype diversity in Munda-speaking populations^14^,^15^ and an independent estimate of an old coalescence age (~65 KYA) of the O2a1-M95 lineage in the Indian AA-speaking populations^10^ suggests an Indian origin followed by a dispersal to Southeast Asia, possibly before the Last Glacial Maximum (LGM, 19.0–26.5 KYA)^19^. This latter hypothesis seems to cope better with the more widely agreed upon costal migration of modern humans from Africa to Asia by way of the Indian subcontinent.

While both theories have certain peculiar merits, neither has dealt well with the large discrepancy of the estimated ages of the O2a1-M95 lineage from different studies. One explanation for the marked differences in the estimate may be limited samplings of the AA speakers in India and/or different genotyping approaches^10^,^12^. Fortunately, a recent study with a more extensive sampling of the AA speakers in India and a few samples from Southeast Asia^9^ has clarified some of these inconsistencies. Through a genome-wide screening of 610K autosomal sequence variations and uniparental loci, Chaubey *et al.* demonstrated an older coalescent time (average 22.4 ± 4.9 KYA) of the O2a1-M95 lineage in Southeast Asia than that in India (average 15.9 ± 1.6 KYA), lending greater credence to the proposed westward migration of the AA speakers from Southeast Asia to India. Chaubey *et al.* also proposed a sex-specific admixture of the AA-speaking immigrants with local India populations by showing a different pattern in the mtDNA lineage^9^.

Despite the data contributions from Chaubey *et al.* and numerous other studies on AA speakers, AA populations from MSEA and southern China continue to be under- sampled and represented. Similarly, no other southern populations have been included in these analysis to date, in spite of the high frequency of O2a1-M95 in certain populations, such as among Daic-speaking populations that have a ~45% frequency^20^,^21^,^22^,^23^. Complicating these oversights, existing genomic analysis also suffers from some deficiencies. For example, the Illumina Human Hap 610K Chips were developed by covering sequence variations identified in limited world populations, which in turn limits its power to detect genetic relationships among the hypothetically ancient AA populations. Given the sampling, methodology and technical limitations inherent in the existing literature, basic questions—where did the O2a1-M95 carrying AA-speakers originally emerge, or when did it begin expanding into Asia—remain unanswered.

In this study, we collected samples of 21 AA-speaking populations from Cambodia, Thailand and southern China (totally, 646 males)(Fig. 1). For individuals belonging to the O2a1-M95 lineage (343 of the 646)^18^, we conducted genotyping of 16 Y-STRs. We also collected published data of the O2a1-M95 carriers from 107 populations (2,510 O2a1-M95 out of 7,693 male individuals in total) covering all the geographic distributions of the AA speakers as well as the other major language families in eastern Asia and India. To date, this data marks the most comprehensive collection of data of O2a1-M95 diversity. Our analysis showed that the O2a1-M95 lineage initially originated in the southern part of eastern Asia among the Daic-speaking populations around 20–40 KYA, followed by a southward dispersal to the heartland of MSEA ~16 KYA, and then a westward migration to India ~ 10 KYA. Furthermore, analysis of more than 20,000 mtDNA sequences, including these AA populations and other Asian populations, demonstrated that the maternal lineage has a different pattern from the Y-chromosome for these AA populations, supporting the proposed sex-biased admixture of the AA immigrants with local people in the Indian subcontinent.

## Results

### High O2a1-M95 frequencies in the AA populations from MSEA and southern China

The O2a1-M95 lineage was reported to be highly prevalent in some AA populations in India, e.g., as high as 67.53% and 74.00% respectively in Munda and Mon-Khmer populations^9^,^10^. We observed high O2a1-M95 frequencies in AA populations not analyzed in previous studies from Cambodia (70.67%), Thailand (52.51%) and Southern China (30.00%) (Fig. 2A, Table 1 and supplementary Table S2)^10^,^11^,^12^,^15^. In the Andaman-Nicobar islands, O2a1-M95 was also widespread (~45.18% on average) and is fixed (100%) in several populations, such as the Shompen and Onge^9^,^10^, likely due to a strong bottleneck effect in these island populations, which is reflected in other major Y-chromosome lineages (e.g. DE-YAP and O3-M22)^24^,^25^,^26^. (Fig. 2A and supplementary Table S2). Consistent with previous results, the collective data shows that O2a1-M95 lineage is dominant in almost all AA populations, including those from MSEA and southern China, making it an informative genetic marker for tracing the patrilineal prehistory of the AA populations.

### Dating the O2a1-M95 lineages of different Asian populations based on Y-STRs variations

**Figure 1:**
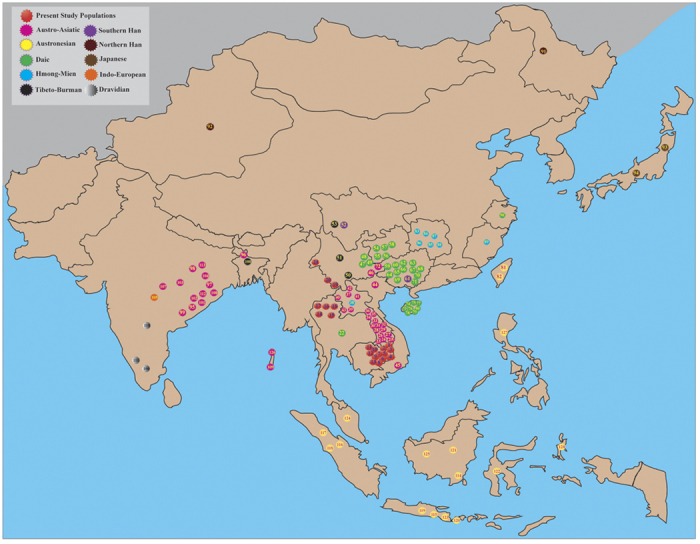
Geographic locations of the studied populations in Asia that contain the O2a1-M95 lineage. Populations are color-coded based on their language families. The figure was modified from our previous report^43^ using Microsoft Powerpoint 2011 (Microsoft Corporation, USA).

Previous studies have sampled few AA populations from MSEA and Southern China^9^,^10^,^12^. To fill the sampling gap, we sampled a wide range of AA-speaking populations from Cambodia, Thailand and southern China^18^ and genotyped 16 Y-STRs loci for those samples belonging to the O2a1-M95 lineage (Fig. 1, Table 1 and supplementary Table S1). Integrating these samples with the previous data, we dated the O2a1-M95 lineages among different regional populations (Fig. 3, supplementary Tables S3 and S4) and observed that the O2a1-M95 lineage has the oldest time of most recent common ancestor (TMRCA) among the populations in the southern part of mainland China and Taiwan (~20–40 KYA), most of which are Daic speaking (Fig. 3, Supplementary Tables S3 and S4). The average TMRCA for these Daic and Austronesian populations from southern China is ~30 KYA, markedly older than those in MSEA (~16 KYA), India (~10 KYA) or Island Southeast Asia (ISEA, ~11 KYA) (Fig. 3, supplementary Tables S3 and S4). The estimated coalescence ages for the AA speakers from MSEA, ISEA and India are similar to those reported by Chaubey *et al.*^9^. At the same time, the estimated ages of O2a1-M95 lineages in the Daic populations was consistent with the estimated ages of its sister lineages (O3-M122 and C-M130) in the same geographic regions^3^,^27^, supporting the proposed antiquity of the Daic populations. These lines of evidence suggest that the O2a1-M95 lineage initially originated in the Daic populations living in southern China, prior to a southward expansion to MSEA and later migrations to India and ISEA after the LGM (19.0–26.5 KYA)^19^.

### Comparison of haplotype diversity of the O2a1-M95 lineages among different geographic populations

**Figure 2:**
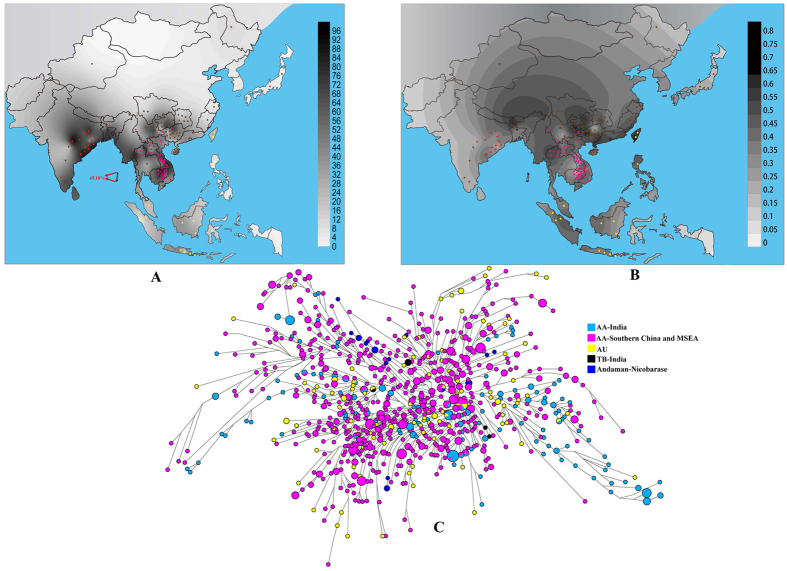
Frequency distribution, Uh diversity and phylogenetic structure of the O2a1-M95 lineages among Asian populations. Contour map shows the frequency (**A**) and Y-STRs Uh diversity (**B**) of lineage O2a1-M95 in Asia. Colored dots indicate the geographic locations of the analysed populations that correspond with Fig. 1; Bars indicate the frequency and Uh diversity spectrum respectively. (**C**) Phylogenetic network of Y-STRs haplotypes among O2a1-M95 populations generated from the following 14 Y-STRs: DYS19, DYS389 I, DYS389II, DYS390, DYS391, DYS392, DYS393, DYS437, DYS438, DYS439, DYS448, DYS458, DYS635 and GATA H4; Circles size is proportional to the number of samples. The contour maps were generated using Surfer10 (Golden Software Inc., Golden, USA), and the network was constructed using the Network package 4.6.1.3 (www.fluxus-engineering.com).

In line with the estimated TMRCAs, the unbiased Y-STRs haplotype diversity of the O2a1-M95 lineage are the highest in populations from southern China (~0.5017 on average), particularly among the Daic populations, followed by those in MSEA (~0.3858), ISEA (~0.3680) and then India (~0.3168) (Fig. 2B and supplementary Table S5), which together match the proposed migratory routes from southern China to MSEA, and then to ISEA and India. We further calculated the pairwise genetic distances measured by *Fst* (supplementary Table S6) and constructed an un-rooted neighbor-joining (NJ) tree based on the Y-STRs variations that showed populations clustered primarily along their respective language families and not by geographic regions. This tree structure suggests a within language family genetic affinity, though there were several interesting exceptions (Fig. 4). The AA populations from India clustered with the AA populations from Cambodia, not with the Dravidian and Indo-European speakers from India. This grouping strongly supports the hypothesized shared genetic ancestry among the AA populations, consistent with the previous observation by Chaubey *et al.*^9^. We also observed a lack of clear geographic clustering in the Y-STRs based phylogenetic network of O2a1-M95 (Fig. 2C), likely due to continuous gene flows among the regional AA speakers^9^.

Interestingly, our analysis departed from several previous observations that had found a clear divergence of the Andaman-Nicobar Island populations from the other AA speakers^9^. Here, we detected shared Y-STRs haplotypes in these isolated island populations with some MSEA populations (Fig. 2C), which may not have been apparent in earlier studies due to a generalized under representation of MSEA populations.

### mtDNA diversity suggests a sex-biased migration of AA-speakers from MSEA to the Indian subcontinent

To check the maternal side of the AA populations, we collected 21,470 mtDNA sequences from 545 populations distributed in East Asia, Southeast Asia and South Asia (supplementary Table S7) and analyzed the patterns of mtDNA diversity. Compared with the dominant occurrence of the O2a1-M95 lineage (65.53% on average) and the high frequency (e.g., 44.57%) of other East Asian specific lineages (NO, N, O, P and Q, supplementary Table S8) in the South Asia populations, we found only ~16.46% mtDNA sequences belonging to the East Asian specific lineages (A, B, C, D, F, G, M9 and M12) in South Asia (supplementary Figures S1, S2 and supplementary Table S9). PCA analysis using mtDNA haplotype frequencies indicated a clustering pattern of geographic locations and not language families, which is different from that of the Y-chromosome data. For example, AA and TB populations from India clustered with Dravidian and Indo-European populations from India and not the other AA populations from southern China and Southeast Asia (Fig. 5). This discrepancy supports the notion that the prehistoric migration of the AA-speakers from MSEA to India was likely sex-biased, confirming the hypothetical sex-biased admixture of the India AA populations posited by Chaubey *et al.*^9^.

## Discussion

Throughout the previous studies, the two primary competing conceptions on the origin and prehistoric migratory pattern of the AA populations have left considerable debate, in part due to not including a wider geographic sampling. Here, we tested these rival hypotheses by systematically collecting AA samples from MSEA and southern China, and observed high frequencies of the O2a1-M95 lineages across all the studied AA populations. This broader survey confirmed that this Y-chromosome lineage represents a genetic signature of all AA populations, and can serve as an effective genetic marker for tracing the prehistoric movements and origins of these populations.

The Y-chromosome data collected in this study does not support an Indian origin of the O2a1-M95 lineage, but instead shows that O2a1-M95 carriers in India originated in southern China and then migrated from MSEA to India around 10 KYA after the LGM. Moreover, our broader analysis that included Daic speaking populations from southern China showed that this population possessed the most diversified O2a1-M95 lineage with an average coalescence age of ~30 KYA, making it the oldest of all known O2a1-M95 carrying populations, and thereby supporting an initial origin of this Y-chromosome lineage in Daic speakers who migrated southward to MESA and a later westward to India ~10 KYA. During the preparation of this manuscript, Arunkumar *et al.* published a similar analysis of O2a1-M95 in Asian populations^28^, and their data also favored an east-to-west migration although the estimated age of migration was much younger than ours due to different mutation rates and methods used for age estimation. Our analysis of mtDNA diversity suggests that after dispersal to India, the O2a1-M95 carrying populations widely absorbed the local maternal gene pool. In contrast to the well-known earliest migration of modern humans from Africa to eastern Asia by way of the Indian subcontinent, our data illustrates a back wave and sex-biased migration of the AA speakers from MSEA to India after the LGM, hinting at a far more complex prehistory of Paleolithic human populations.

## Materials and Methods

### Genotyping and data collection

For the 343 male samples that belong to the O2a1-M95 lineage from our previous study (Table 1)^18^, we genotyped 16 Y-STRs (DYS19/394, DYS388, DYS389 I, DYS389 II, DYS390, DYS391, DYS392, DYS393, DYS437, DYS438, DYS439, DYS448, DYS458, DYS461, DYS635 and GATA H4) with the methods described previously^29^,^30^. DYS389I (DYS389cd) was subtracted from DYS389II and renamed 389ab because DYS389II contains the repeat number of DYS389I. To dissect the origin and migratory patterns of the O2a1-M95 lineage, we collected all available O2a1-M95 Y-STRs data, which covers 107 geographic populations (up to 2,510 samples carrying O2a1-M95) from East Asia, Southeast Asia and South Asia^9^,^12^,^20^,^21^,^22^,^23^,^24^,^26^,^31^,^32^,^33^,^34^,^35^,^36^,^37^,^38^ (Fig. 1, supplementary Tables S1 and S2).

### Data analysis

**Figure 3:**
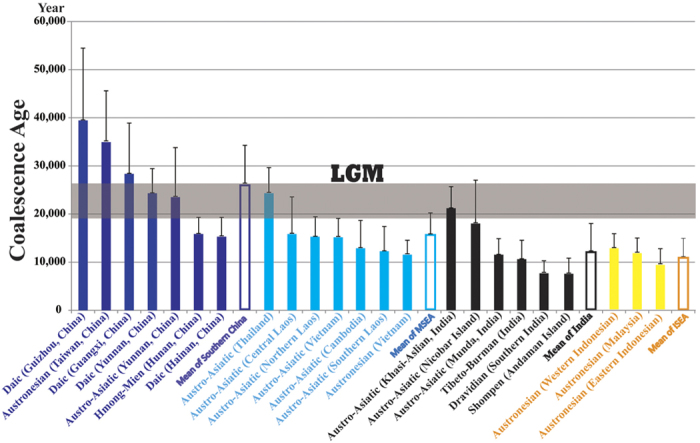
Comparison of coalescence ages of the O2a1-M95 lineages among diffenent geographic populations. The age of each geographic or linguistic group was calculated by taking the average of respective populations from supplementary Table S3.

**Figure 4:**
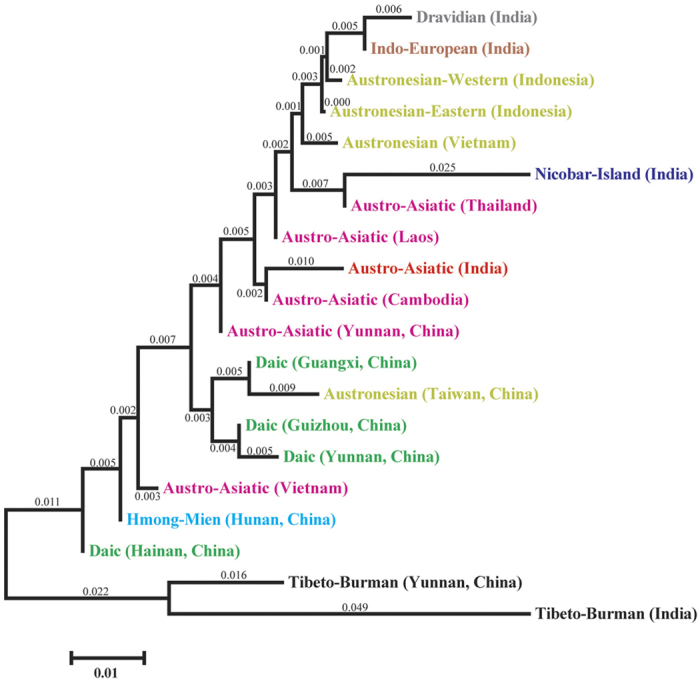
NJ-tree constructed of Y-STRs variations among different language family populations. Different linguistic families are shown using different colors. Branch length values are indicated above the branch.

**Figure 5:**
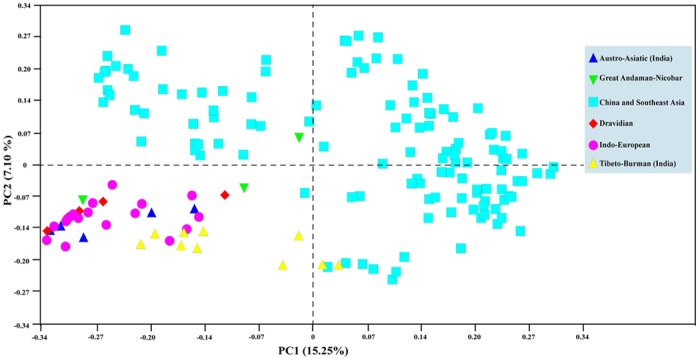
Map of principal component analysis (PCA) among Asian populations. Populations of East Asia and South Asia were grouped respectively by geograpghic region and language family. AA and TB-speaking populations closely clustered with DR anf IE populations in the lower left. The first and the second components explain 15.25% and 7.10% of the genetic variance, respectively.

We estimated the time of most recent common ancestor (TMRCA) of the O2a1-M95 lineage using Y-STRs variation in each population as described previously, with a 25-year generation time and a mutation rate of 6.9 × 10^−4^ 12,39 (supplementary Table S3). For comparison, when calculating the ages we used three sets of loci for each population: a) the actual number of loci in the corresponding references, b) a 7-loci set (DYS19, DYS389 I, DYS389 II, DYS390, DYS391, DYS392 and DYS393) and c) a 6-loci set (DYS19, DYS389 I, DYS390, DYS391, DYS392 and DYS393), and the results from different calculations are very similar for most populations (supplementary Table S4). The mean TMRCAs of a geographic region are the average of its populations (Fig. 3). We also estimated the unbiased haplotype diversity of every population using GenAlEx 6.3. When estimating the age and diversity, O2a1-M95 populations with less than 10 samples were either excluded or merged to other closely related populations. In total, the coalescence ages and diversity of the O2a1-M95 lineages from 105 Asian populations were calculated (supplementary Tables S3 and S5).

A median-joining network, resolved with the MP algorithm, was constructed using the Network package 4.6.1.3 (www.fluxus-engineering.com). The O2a1-M95 variance isofrequency maps based on frequency and unbiased haplotype diversity were generated using Surfer10 (Golden Software Inc., Golden, USA), following the Kriging procedure. Average number of pairwise difference of Y-STRs for the studied populations was calculated using the Arlequin 3.5^40^, and NJ-tree was constructed with MEGA 6.0^41^. We performed principal component analysis (PCA) based on the frequencies of mtDNA haplogroups according to the method developed by Richards *et al.*^42^ with MVSP 3.13.

To compare the paternal and maternal gene pool between populations from East Asia and South Asia, we analyzed ~21,470 mtDNA sequences among these populations published previously (Supplementary Table S7).

## Additional Information

**How to cite this article**: Zhang, X. *et al.* Y-chromosome diversity suggests southern origin and Paleolithic backwave migration of Austro-Asiatic speakers from eastern Asia to the Indian subcontinent. *Sci. Rep.* **5**, 15486; doi: 10.1038/srep15486 (2015).

## Supplementary Material

Supplementary Information

Supplementary Tables

## Figures and Tables

**Table 1:** Sampled populations from MSEA and southern China.

NO.	Population	Region	Location	Linguistic Family	Sub-Branch	N	O2a1-M95 Counts	%
1	Brao	Cambodia	Ratanakri	Austro-Asiatic	West Bahnaric	37	24	64.86
2	Jarai	Cambodia	Ratanakri	Austronesian	Chamic	45	34	75.56
3	Kachac	Cambodia	Ratanakri	Austro-Asiatic	North Bahnaric	17	13	76.47
4	Khmer	Cambodia	Kratie	Austro-Asiatic	Khmer	34	18	52.94
5	Kravet	Cambodia	Ratanakri	Austro-Asiatic	West Bahnaric	24	12	50.00
6	Kreung	Cambodia	Ratanakri	Austro-Asiatic	West Bahnaric	22	14	63.64
7	Kuy	Cambodia	Stung Treng	Austro-Asiatic	Katuic	37	34	91.89
8	Lao	Cambodia	Stung Treng	Daic	Kadai	27	14	51.85
9	Lun	Cambodia	Ratanakri	Austro-Asiatic	West Bahnaric	13	12	92.31
10	Mel	Cambodia	Kratie	Austro-Asiatic	Monic	19	15	78.95
11	Phnong	Cambodia	Kratie	Austro-Asiatic	South Bahnaric	26	20	76.92
12	Stieng	Cambodia	Kratie	Austro-Asiatic	South Bahnaric	12	8	66.67
13	Tompoun	Cambodia	Ratanakri	Austro-Asiatic	South Bahnaric	51	37	72.55
14	Kraol	Cambodia	Ratanakri	Austro-Asiatic	South Bahnaric	1	1	100%
14	Blang	Thailand	Chiang Rai	Austro-Asiatic	Waic	7	5	71.43
15	Htin	Thailand	Nan	Austro-Asiatic	Mal-Phrai	35	30	85.71
16	Lawa	Thailand	Chiang Mai	Austro-Asiatic	Waic	41	14	34.15
17	Palaung	Thailand	Chiang Mai	Austro-Asiatic	Palaung-Riang	16	3	18.75
18	Mon	Thailand	Chiang Mai	Austro-Asiatic	Monic	2	0	0
19	Bulang	China	Yunnan	Austro-Asiatic	Waic	55	17	30.91
20	Wa	China	Yunnan	Austro-Asiatic	Palaung-Riang	57	5	8.77
21	De’ang	China	Yunnan	Austro-Asiatic	Waic	68	13	19.12
					**Total**	646	343	53.10
