# Supplementary material for: Y-chromosome diversity suggests southern origin and Paleolithic backwave migration of Austro-Asiatic speakers from eastern Asia to the Indian subcontinent
Source: Sci Rep. 2015 Oct 20;5:15486. doi: 10.1038/srep15486 (PMC4611482; doi:10.1038/srep15486)

Supplementary Information

Y-chromosome diversity suggests southern origin and Paleolithic backwave migration of Austro-Asiatic speakers from eastern Asia to the Indian subcontinent

Xiaoming Zhang[1,9], Shiyu Liao[2,9], Xuebin Qi[1,9], Jiewei Liu[1,8], Jatupol Kampuansai[5], Hui Zhang[1], Zhaohui Yang[3,4], Bun Serey[6], Tuot Sovannary[6], Long Bunnath[6], Hong Seang Aun[6], Ham Samnom[7], Daoroong Kangwanpong[5], Hong Shi[3,4]* & Bing Su[1,4]*

Figure S1. Network of mtDNA lineages under the macro-haplogroup M in East Asia and South Asia populations. The mtDNA data is collected from the references in supplementary Table S7.

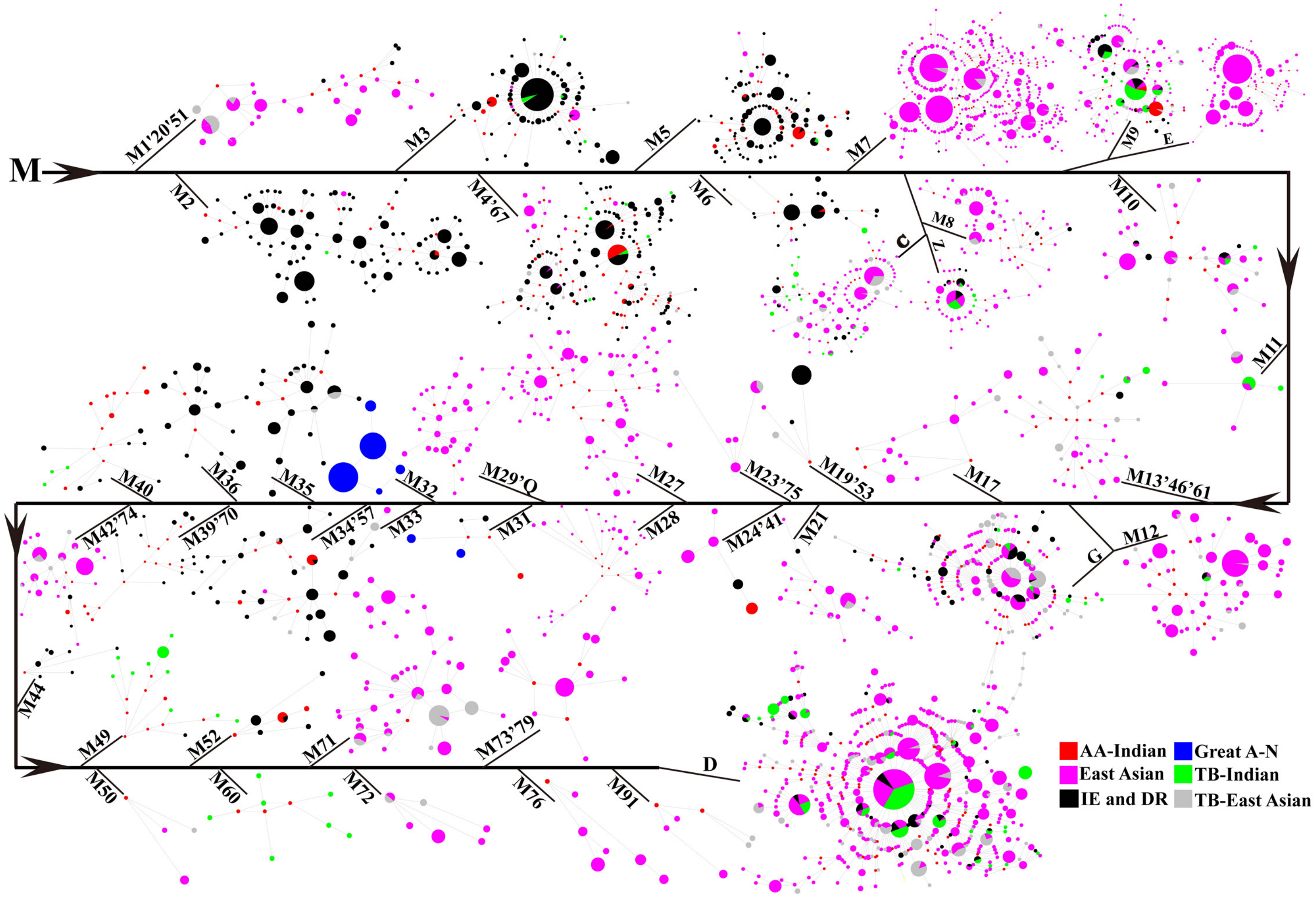

Figure S2. Network of mtDNA lineages under the macro-haplogroup N in East Asia and South Asia populations. The mtDNA data is collected from the references in supplementary Table S7.

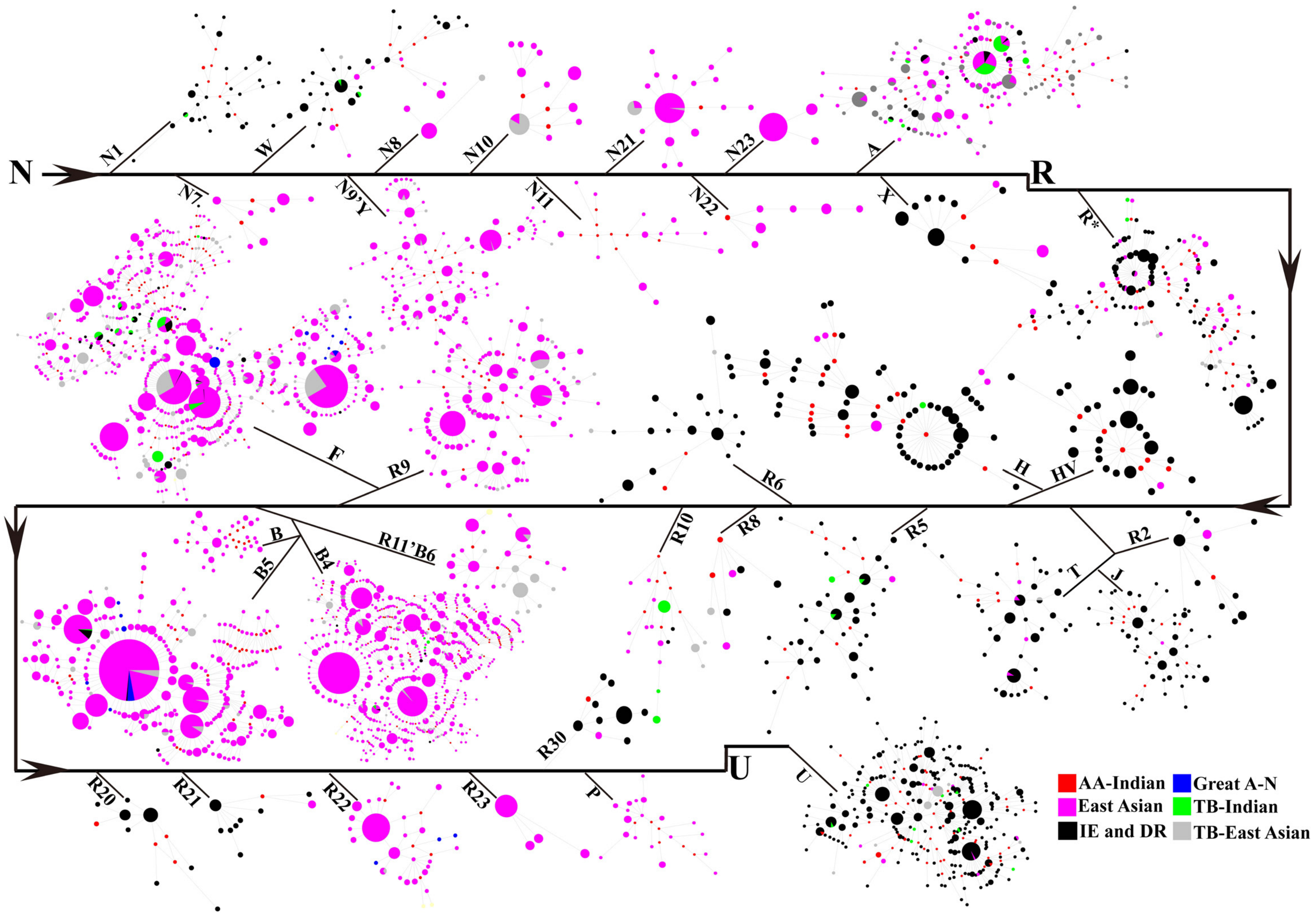

Supplement: Supplementary Information [file srep15486-s1.pdf]
